# Richer fusion network for breast cancer classification based on multimodal data

**DOI:** 10.1186/s12911-020-01340-6

**Published:** 2021-04-22

**Authors:** Rui Yan, Fa Zhang, Xiaosong Rao, Zhilong Lv, Jintao Li, Lingling Zhang, Shuang Liang, Yilin Li, Fei Ren, Chunhou Zheng, Jun Liang

**Affiliations:** 1 High Performance Computer Research Center, Institute of Computing Technology, Chinese Academy of Sciences, Beijing, China; 2 University of Chinese Academy of Sciences, Beijing, China; 3 Department of Oncology, Peking University International Hospital, Beijing, China; 4 Institute of Computing Technology, Chinese Academy of Sciences, Beijing, China; 5 Department of Pathology, Peking University International Hospital, Beijing, China; 6 Xingtai People’s Hospital, Hebei, China; 7 College of Computer Science and Technology, Anhui University, Hefei, China

**Keywords:** Pathological image, Electronic medical record, Multimodal fusion, Breast cancer classification, Convolutional neural network

## Abstract

**Background:**

Deep learning algorithms significantly improve the accuracy of pathological image classification, but the accuracy of breast cancer classification using only single-mode pathological images still cannot meet the needs of clinical practice. Inspired by the real scenario of pathologists reading pathological images for diagnosis, we integrate pathological images and structured data extracted from clinical electronic medical record (EMR) to further improve the accuracy of breast cancer classification.

**Methods:**

In this paper, we propose a new richer fusion network for the classification of benign and malignant breast cancer based on multimodal data. To make pathological image can be integrated more sufficient with structured EMR data, we proposed a method to extract richer multilevel feature representation of the pathological image from multiple convolutional layers. Meanwhile, to minimize the information loss for each modality before data fusion, we use the denoising autoencoder as a way to increase the low-dimensional structured EMR data to high-dimensional, instead of reducing the high-dimensional image data to low-dimensional before data fusion. In addition, denoising autoencoder naturally generalizes our method to make the accurate prediction with partially missing structured EMR data.

**Results:**

The experimental results show that the proposed method is superior to the most advanced method in terms of the average classification accuracy (92.9%). In addition, we have released a dataset containing structured data from 185 patients that were extracted from EMR and 3764 paired pathological images of breast cancer, which can be publicly downloaded from http://ear.ict.ac.cn/?page_id=1663.

**Conclusions:**

We utilized a new richer fusion network to integrate highly heterogeneous data to leverage the structured EMR data to improve the accuracy of pathological image classification. Therefore, the application of automatic breast cancer classification algorithms in clinical practice becomes possible. Due to the generality of the proposed fusion method, it can be straightforwardly extended to the fusion of other structured data and unstructured data.

## Background

Cancer is a critical global public health problem. For women, the three most common cancers are breast cancer, lung cancer, and colorectal cancer, which together account for half of the new diagnoses in 2019. Breast cancer alone accounts for 30% of all new cancer diagnoses [1]. Even with the rapid development of medical science, the analysis of pathological images is still the gold standard for breast cancer diagnosis [2]. However, the complexity of pathological images and the dramatic increase in workloads make this task time consuming, and the results may be affected by the pathologist's subjectivity. Faced with these challenges, it is urgent to propose an accurate and automatic analysis method for breast cancer diagnosis.

In recent years, deep learning methods have achieved outstanding results in the field of computer vision, which has inspired many researchers to transfer this method to pathological image analysis. In spite of this, the classification accuracy of benign and malignant breast cancer using only single-mode pathological image data does not meet the requirements of clinical practice.

Although pathological images alone do not yield good breast cancer classification results, pathological images provide a rich environment to integrate electronic medical record (EMR) data, making it possible to fully integrate multimodal data and discover new information after fusion. In particular, the original pathological images are high dimensional information. It requires less human effort to obtain, but it contains large amounts of information that is potentially unrecognizable to humans. The clinical information extracted from EMR is low dimensional, but this information is usually the summary of doctors' professional knowledge and long-term experience, which can provide more guidance for diagnoses.

**Fig. 1:**
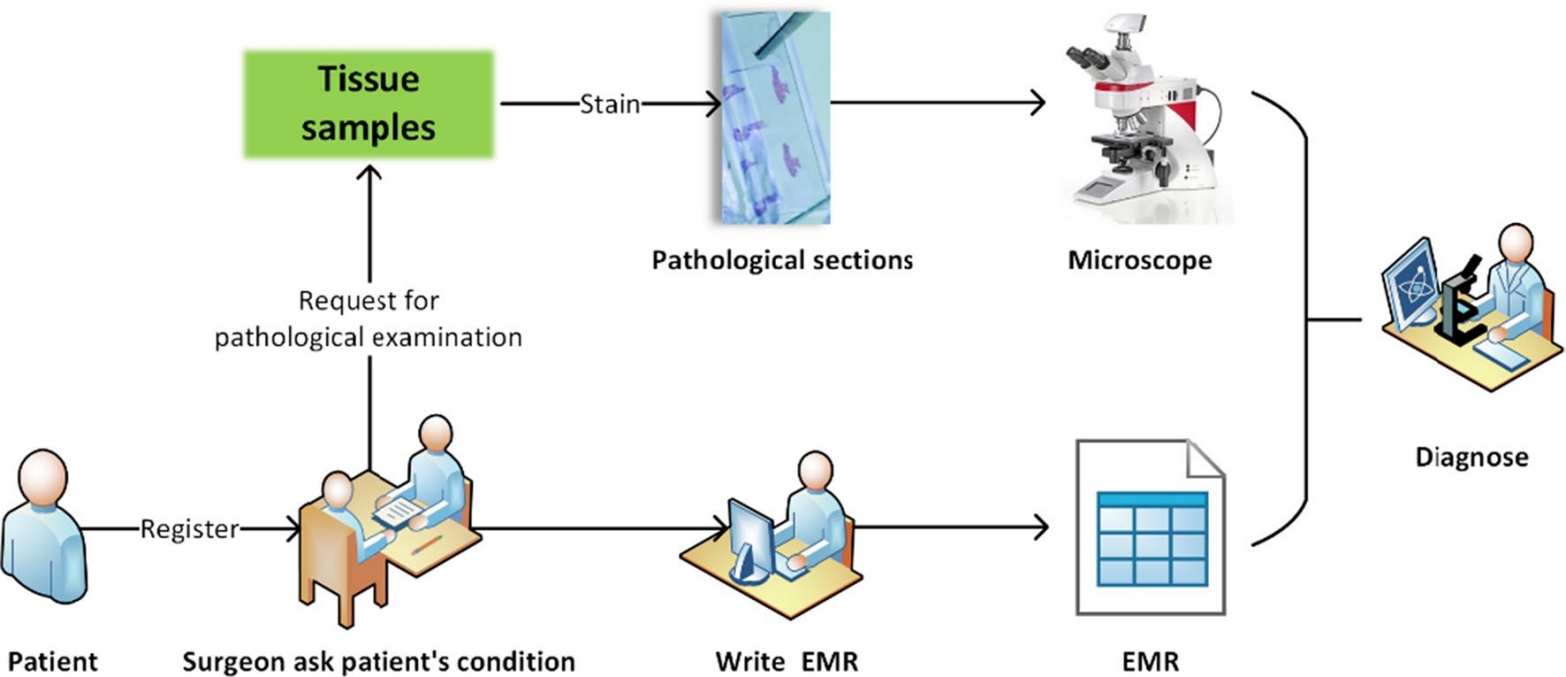
A simple introduction to pathological diagnosis workflow in the hospital. When patient came to the hospital for treatment, they first register with the corresponding department (e.g., breast surgery). The surgeon then determines whether the patient needs a pathological examination based on the information obtained by asking about the patient's condition and the information obtained from the surgical examination, and records the information into the EMR system. Finally, the pathologist makes a diagnosis by carefully reading the patient's pathological images and repeatedly combining the information from the EMR

Therefore, we integrate the analysis of different data modalities to simulate diagnostic tasks in clinical practice. From the perspective of multimodal data fusion, we try to combine pathological images with structured EMR data to further improve the diagnostic accuracy for breast cancer. This approach is also consistent with the actual scenario in which the pathologist reads pathological images for diagnosis, as shown in Fig. 1. When reading a pathological image, the pathologist will repeatedly refer to the relevant information in the patient's EMR as a priori information and guidance until the final diagnosis.

There is almost no literature on the classification of breast cancer using both EMR data and pathological images. However, the multimodal fusion approach has achieved outstanding results in other medical fields (text, images, and genomics). Nevertheless, there are still some problems, such as the feature representation of images not being rich enough and information fusion being insufficient. In particular, the loss of high-dimensional information before data fusion and the problem of partially missing data are often encountered in real scenarios.

In this paper, we proposed a richer fusion network to integrate multimodal data for breast cancer classification. The main contributions of our work are as follows.

1. To the best of our knowledge, this is by far the first time that structured EMR data and pathological image data have been integrated to classify breast cancer, and the proposed richer fusion network significantly outperforms methods that use any single source of information alone and previous multimodal frameworks.
2. To make pathological image can be integrated more sufficient with structured EMR data, we proposed a method to extract richer multilevel feature representation of the pathological images from multiple convolutional layers. Thus, we can retain the complementary multilevel image information, such as local cell textures and tissue structures.
3. To minimize the information loss for each modality before data fusion, we use denoising autoencoder as a way to increase the low-dimensional EMR data instead of reducing the high-dimensional image data to low-dimensional before data fusion. In this way, there is enough information in each modality before information fusion, which fulfills a prerequisite for more sufficient information fusion. Meanwhile, denoising autoencoder naturally generalizes our method to make the accurate prediction with absent of part structured EMR data.
4. We released a new dataset with pathological images and pairwise multiple types of features that were extracted from EMR for evaluating breast cancer classification algorithms.

## Related work

### Multimodal data fusion

Recently, deep learning has shown excellent performance in the field of natural images, and similarly, breakthroughs have been made in the field of medical images such as pathological image classification [3–5]. Bayramoglu et al. [4] proposed a magnification-independent deep learning method for the breast cancer pathological image classification with a classification accuracy of approximately 83%.

However, such classification accuracy is not sufficient for clinical practice. Inspired by the pathologists' diagnostic process in actual scenarios, the multimodal data fusion approach offers new opportunities. Meanwhile, a distinguishing feature of medical big data is its diversity, which means that large amounts of data are collected in a variety of modalities, including structured and unstructured data, as well as semi-structured data [6]. For example, structured EMR data and unstructured pathological images data. This distinct characteristic of medical data also presents great opportunities.

Moreover, many studies have shown that the performance of multimodal fusion methods is significantly better than using only a single mode. Yao et al. [7] proposed a cancer survival prediction model that integrates cancer protein expression, pathological image and copy number variation using a deep neural network. In addition, Mobadersany et al. [8] considered the interaction of multi-omics information and pathological image information for cancer survival prediction. Xu et al. [9] use a hybrid deep neural network for the task of cervical dysplasia classification using multimodal data: Cervigram image and clinical records including age, Potential of Hydrogen (PH) values, Papanicolau (Pap) tests, and Human Papilloma Virus (HPV) tests. The proposed multimodal network can better learn complementary features through backpropagation, and its results are significantly better than those of methods using any single-mode data alone.

Multimodal fusion has achieved outstanding results, but each modality of multimodal objects has its own characteristics, resulting in the complexity of heterogeneous data. Therefore, heterogeneous data present another challenge to the multimodal fusion method.

### High-dimensional and low-dimensional data fusion

High dimensional data (such as images) usually have high dimensional feature representation. In contrast, the dimensions of structured data are inherently low. Whether the high-dimensional data and low-dimensional data can be effectively integrated will have a great impact on the final results.

Information fusion methods can be divided into three categories according to the level of integration: data-level fusion, feature-level fusion, and decision-level fusion [10]. Generally, the smaller the information loss of each modality before fusion is, the more sufficient the information fusion is, and the better the final fusion result is. From existing research, the correctness of this view is also proved, especially that feature-level fusion is better than decision-level fusion [11]. This finding indicates that the information for each modality, especially the high-dimensional modalities with rich information, should be as complete as possible before fusion and then reduced to the desired level after the initial fusion of the multimodal data. Therefore, the fusion is sufficient to effectively capture the complex associations of multimodal heterogeneous data.

At present, most data fusion is image and image or image and text [12, 13]. However, these data are all of high dimensions, and few early studies involve the fusion of low-dimensional structured data and high-dimensional unstructured data. Xu et al. [9] first reduced high-dimensional Cervigram images to low-dimensional and then merged them with low-dimensional clinical records (i.e., age, PH values, Pap tests, and HPV tests). This method has achieved good results. However, this method loses substantial image information before fusion, which makes the fusion insufficient.

### Richer feature representation of image

The precondition for multimode fusion is the feature extraction of each single-mode data. Current deep learning methods enable the learning of very good feature representation from unstructured data, such as images. This also makes the deep learning methods achieve good results in tasks such as image classification, edge detection, and semantic segmentation.

In the semantic segmentation and edge detection tasks, the performance of the algorithm is greatly improved by the use of richer multilevel features. Sermanet et al. [14] classified digits of real-world house numbers using the traditional convolutional neural network architecture augmented with multi-stage features. Liu et al. [15] proposed a method to perform image-to-image prediction by holistically merging all meaningful convolution features and making full use of the multiscale and multilevel information of objects. Bertasius et al. [16] presented a multi-scale bifurcated deep neural network for top-down contour detection. To capture the representation of hierarchical information, these networks extract features at multilevel convolutional layers. Xie et al. [17] proposed a holistically nested edge detection network to learn richer hierarchical representation, which are effective for alleviating the ambiguity in edge and object boundary detection.

However, different tasks have different characteristics, which make it necessary to select the most appropriate feature representation that is required for a particular task. Unlike the image semantic segmentation and edge detection task, which needs to extract richer multilevel feature representation, image classification needs to extract more abstract low-dimensional feature representation. Therefore, the image classification task repeated down-sampling combinations that were performed in successive layers of CNNs. Then, the final feature map is flattened, and the dimension is reduced to the required classification dimension through the fully connected layer [18]. This is a process of information compression, but also a process of information loss.

For the task of integrating multimodal data for classification, the key factor is whether the fusion between different modalities is sufficient. Therefore, we should not extract abstract low-dimensional feature representation in the way of classification task, but rather, we should extract richer multilevel feature representation in the way of image semantic segmentation and edge detection tasks. In this way, each modality can learn enough feature representation before fusion to provide a fertile environment for full multimodal fusion.

### Denoising autoencoder

Autoencoders [19] are a type of unsupervised machine learning algorithm and are widely used for data dimensionality reductions. However, in the existing research, it is rare to use autoencoder or its variant to improve the dimension of data. The denoising autoencoder [20] is based on the autoencoder, adding noise to the training data, and the output label is still the original sample without noise. The autoencoder must learn to remove noise to obtain raw input characteristics that are not corrupted by noise. Thus, this forces the encoder and decoder to learn more robust feature representation of the original input data. In actual training, the method of adding noise can also be artificial to make the original input data incomplete. Inspired by denoising autoencoders, Ngiam et al. [21] proposed training the bimodal (audio and video) autoencoder using a noisy dataset. Specifically, these authors proposed to add examples that have zero values for one of the input modalities and original values for the other input modality but still train the bimodal autoencoder to reconstruct both modalities.

## Dataset

It is well known that high-quality annotated datasets play an important role in deep learning applications. An important reason for the significant progress that is made in the field of computer vision is the publicly available benchmark datasets, such as ImageNet [22], that can be used for large scale visual recognition. Inspired by these datasets, pathological image researchers eventually began to follow this trend, releasing well-annotated benchmark datasets such as BreaKHis [3] for histopathological image classification.

However, there is still no publicly available matching clinical EMR data and pathological image dataset for breast cancer research. In this work, we collaborated with Peking University International Hospital to release a new benchmark dataset containing pathological images and pairwise multiple types of attributes that were extracted from clinical EMR for benign and malignant breast cancer classification. We named it PathoEMR dataset (Pathological EMR Dataset). The overall description of the PathoEMR dataset is shown in Table 1.

**Table 1:** The overall description of the PathoEMR dataset

Description	Value
Number of medical records	185 (82 benign, 103 malignant)
Number of pathological images	3764 (1332 benign, 2432 malignant)
Size of pathological images	2048 × 1536 pixels
Color model of pathological images	R(ed)G(reen)B(lue)
Memory space of pathological images	3–20 MB
Number of features extracted from each case	29

### Pathological image

**Fig. 2:**
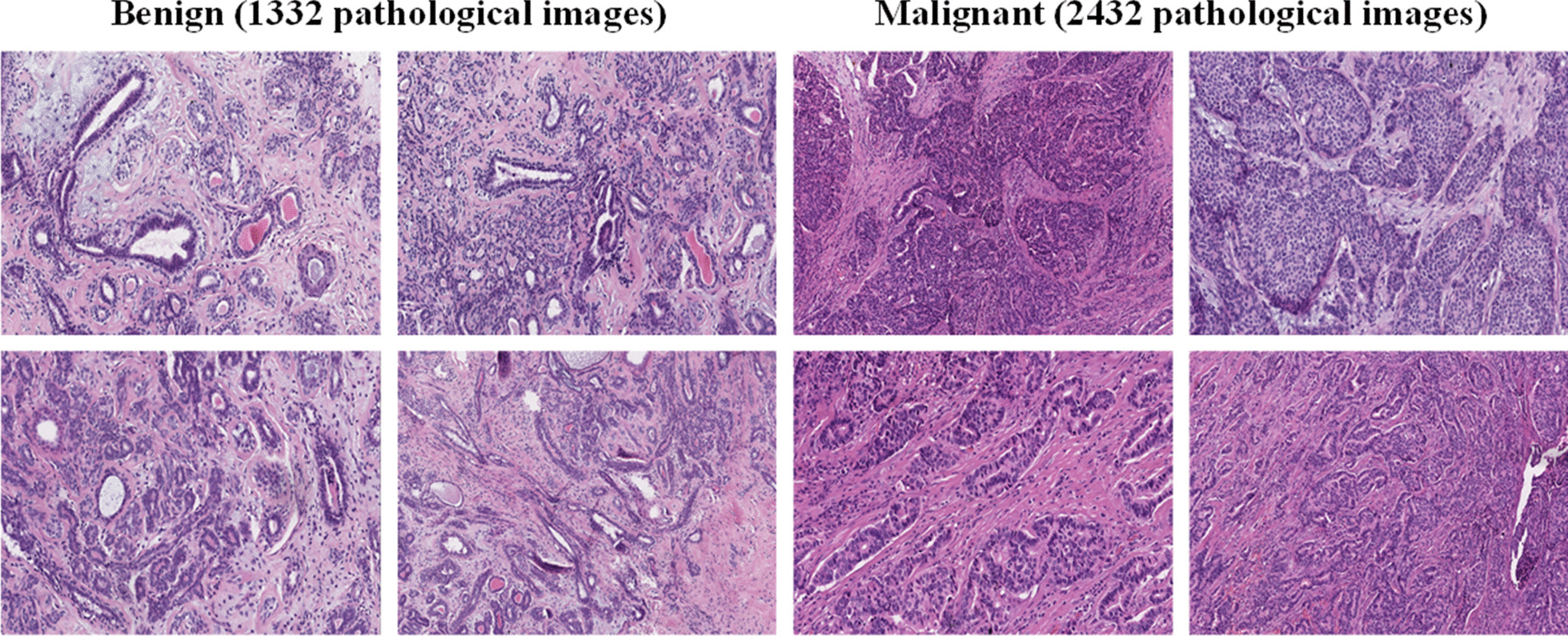
Examples of pathological images in our released dataset

We collected the medical records of 185 breast cancer patients, and for each patient, we collected several whole slide images (WSI) and selectively cropped 2–10 representative image areas from each WSI. In the end, we collected a total of 3764 high resolution (2048 × 1536 pixels) pathological images. All pathological images were obtained using the same equipment at a 100 × or 200 × magnification. Each image is labeled as benign or malignant according to the main cancer type in each image. The annotation of each pathological image was completed by three medical experts, and the annotated images with inconsistent opinions were sent to the pathologists' supervisor for final confirmation. Figure 2 shows an example of the pathological images in the dataset and summarizes the image distribution. The institutional review committee of Peking University International Hospital approved the study, and all the related data are anonymous. All pathological images were obtained using a Leica Aperio AT2 slide scanner from March 2015 to March 2018.

### Structured EMR data

After discussion with the pathologists, we extracted 29 representative features from the EMR. These features are closely related to the medical theory on the diagnosis of breast cancer, and these structured data are used to describe the patients' condition. Specifically, these 29 features include age, gender, disease course type, personal tumor history, pectoral muscle adhesion, family tumor history, orange peel appearance, prophase treatment, breast deformation, neoadjuvant chemotherapy, dimple sign, redness and swelling of skin, skin ulcers, tumor, axillary lymphadenectasis, nipple change, nipple discharge, swelling of lymph nodes, tumor position, tenderness, tumor number, tumor size, tumor texture, tumor border, smooth surface, tumor morphology, activity, capsules, skin adhesion and the diagnosis. According to the actual situation, the data have been quantified into specific values. The simple description and value of each feature are shown in Table 2.

**Table 2:** Description of structured EMR data

Feature	Value	Feature	Value
Age: The incidence of breast cancer is rising rapidly after the age of 20	0: Less than 20 1: Between 20 and 40 2: Older than 40	Gender: Male can also get breast cancer	0: Male 1: Female
Disease Course Type: Benign tumors grow slowly, but cancers grow much faster	0: Not mentioned 1: Chronic 2: Acute 3: Hidden	Pectoral Muscle Adhesion	0: No 1: Yes
Personal Tumor History	0: No 1: Yes	Family Tumor History	0: No 1: Yes
Prophase Treatment	0: No 1: Yes	Neoadjuvant Chemotherapy	0: No 1: Yes
Dimple Sign: Tumors invade the suspensory ligament of the breast, it may shrink and pull the skin to form a depression like dimple	0: No 1: Yes	Orange Peel Appearance: The skin thickens and the follicle mouth dilate and sink in	0: No 1: Yes
Redness and Swelling of Skin: Mainly found in inflammatory breast carcinoma	0: No 1: Yes	Skin Ulcers: Advanced cancer may directly invade the skin	0: No 1: Yes
Tumor: Breast tumor	0: No 1: Yes	Breast deformation	0: No 1: Yes
Nipple Change: Patients with abnormal nipple changes, usually manifested as nipple erosion or nipple retraction	0: No change 1: Nipple erosion 2: Nipple retraction	Nipple Discharge: such as bloody nipple discharge	0: No 1: Yes
Axillary Lymphadenectasis (AL): axillary lymph node is the earliest metastasis site of breast carcinoma. The number of metastases can guide treatment plans	0: No AL 1: Movable 2: Lymph node fusion 3: Parasternal lymph node metastasis	Swelling of Lymph Nodes: Benign neoplasm does not metastasize to distant sites	0: No distant metastasize 1: Distant metastasis
Tumor Position: The final detection of breast tumor relies on segmentation of tumor region to a great extent	0: Outer 1: Upper 2: Inner lower 3: Outer lower 4: Central zone	Tumor Number: Most of the breast carcinoma has single tumor in unilateral breast	0: Single-unilateral 1: Multiple-unilateral 2: Bilateral
Tumor Size: The size of the tumor refers to the area of the surrounding tissue infiltrated by the lesion. The measurement should be accurate to millimeters(mm)	0: Less than 20 1: Between 20 and 50 2: Greater than 50	Tumor Texture: Usually the texture of the carcinoma is hard	0: Soft 1: Hard 2: Hard tough 3: Tough 4: Moderate
Tumor Border: Most breast carcinoma shows infiltrative growth with unclear borders. Some can be flat, surface is not smooth	0: Clear 1: Unclear 2: Invasive	Smooth Surface: It's a sign of a benign tumor	0: No 1: Yes
Tumor Morphology: Benign is round or oval, malignant masses exhibit irregularity in shapes	0: Regular 1: Moderate 2: Irregular	Activity: Small tumor has good activity	0: Good 1: Moderate 2: Bad
Capsules: The Benign tumors often have capsules, while malignant tumors have no capsules or incomplete capsules	0: No envelope 1: Incomplete 2: Enveloped	Tenderness: It is mainly found in inflammatory breast carcinoma	0: No 1: Yes 2: Periodicity
Skin Adhesion: A sign of malignancy	0: No; 1: Yes	-	-

## Methods

**Fig. 3:**
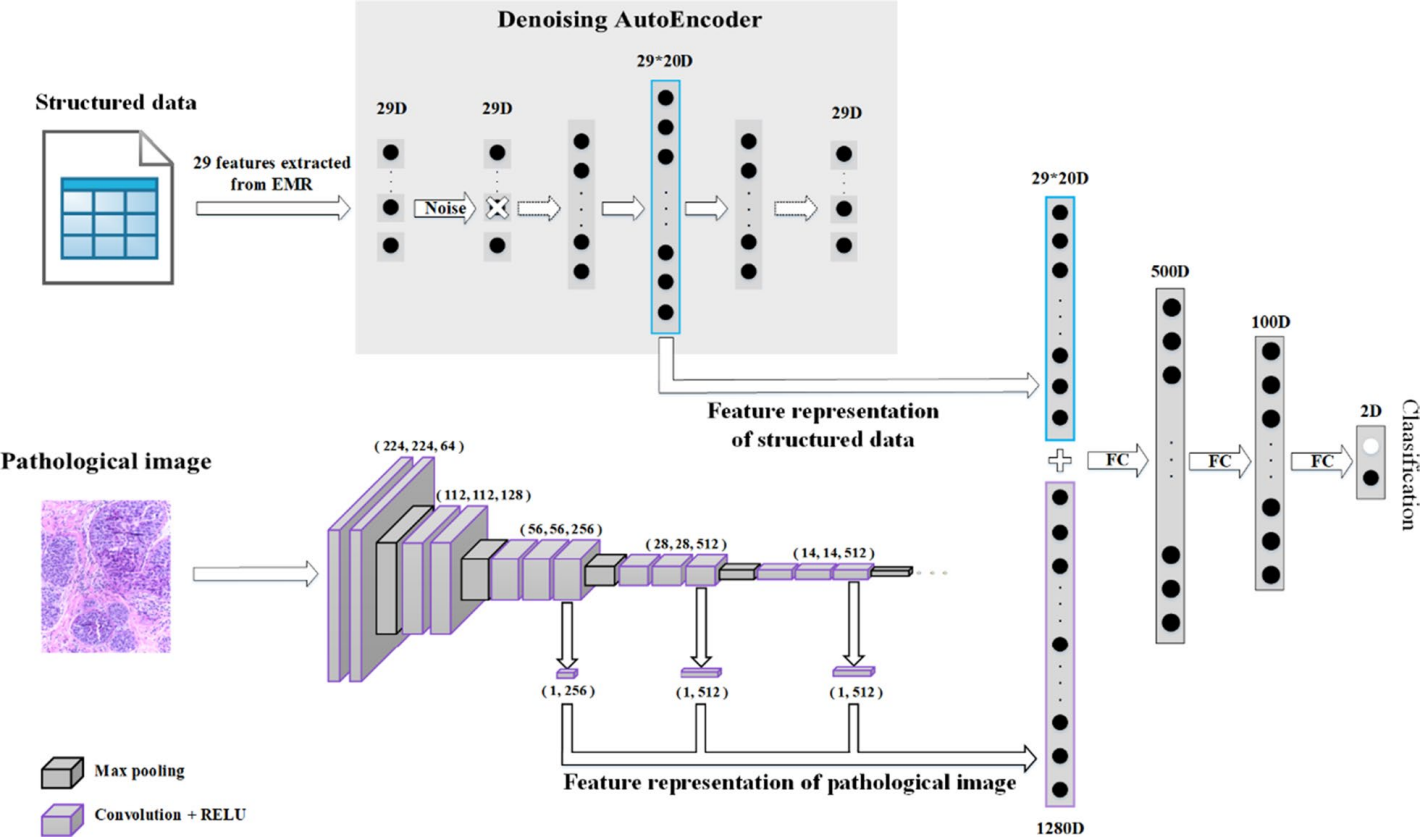
The overall framework of our proposed richer fusion network. (1) In terms of structured data, we extracted 29 representative features from EMR, which are closely related to breast cancer diagnosis in medical theory. We use a denoising autoencoder to increase the 29-dimensional vector to 580 dimensions. Different from the general way of adding noise, we randomly discard a certain feature of the input layer as a way to add noise. (2) In terms of pathological image, the feature maps of the third, fourth and fifth convolution layers were extracted from the VGG16 network (1280-dimensional) as richer feature representation; (3) Finally, the vector of 29D*20 dimensions extracted from the structured data was concatenated with the vector of 1280D dimensions extracted from the pathological images to form a vector of 1860D. This vector then goes through the next three full connection layers to get a classification result between benign and malignant breast cancer. The three full connection layers have 500, 100, and 2 nodes, respectively

In this section, we describe our proposed richer fusion network for breast cancer classification in detail. For the sake of clarity, we first introduce the overall framework of our method, as shown in Fig. 3. Then, we introduce our method from four perspectives: data preprocessing, pathological image feature representation, structured EMR data feature representation and multimodal data fusion.

### Data preprocessing

We first implemented data enhancement. Except for resizing the whole image to 224*224, we randomly extract 40, 20, 10 and 5 patches that are sized 224*224, 512*512, 1024*1024 and 1536*1536, respectively, from the original image of 2048*1536. In addition, we also applied general data enhancement techniques to the image, including random flips, rotations, brightness adjustments, contrast adjustments and so on. Finally, we obtain 3,105,300 pairs of training samples. Notice that a medical record usually corresponds to many pathological images. Therefore, in the training phase, we used the index of pathological images as the guideline, and pairs of pathological images and structured EMR data were sent to the network for training. In other words, there are some duplicate structured EMR data. Aresta et al. [23] concluded that the convolutional neural network (CNN) structure seems to be robust to small color variations of pathological images, and thus, color normalization was not essential to obtaining high performance. Therefore, unlike most of the current applications of deep learning methods to pathological images, we did not implement image normalization preprocessing.

### Pathological image feature representation

Since the cell morphology and tissue structures in pathological images have various scales and high complexity, learning rich hierarchical features is critical for the fusion of multimodal data. CNNs have proven to be effective for feature representation learning. As the convolutional layers are stacked, the features extracted from the CNN gradually become rough. In other words, different convolutional layers extract features with different degrees of abstraction. Inspired by these observations, we are trying to apply multilevel convolutional features to these challenging tasks. The multilevel features we used provide a richer features representation than the features that are extracted only from the last fully connected layer of the CNN. This phenomenon occurs because the multilevel convolutional layer retains additional information, such as local cell textures and tissue structures.

**Fig. 4:**
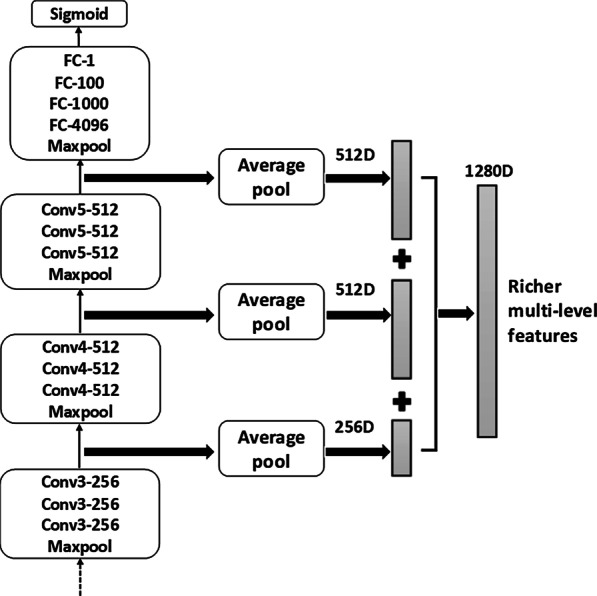
Schematic overview of the richer multilevel feature representation. We use average pooling on the final output of the third, fourth and fifth convolution layers of VGG16 network. Then, we concatenate the three vectors into a 1280D dimensional vector, which is used as the richer multilevel feature representation of the pathological image. The convolutional layer parameters are denoted as “Conv<number of convolutional layers>-<number of channels>”

The VGG [24] is the most commonly used CNN which was developed by the visual geometry group (VGG) at Oxford university, since its powerful recognition and feature extraction capabilities have been proved to be effective on large datasets. Based on a total of 3764 annotated pathological images from our dataset, we first trained the VGG16 network for breast cancer pathological image classification from scratch and then fixed the parameters of the model for the subsequent algorithm. The top of the VGG16 network structure that we used is shown in Fig. 4. We also tried to fine-tune the pretrained model to train the VGG16. When the amount of data is small, the pretraining model can indeed converge faster and achieve good results. However, the gap between natural images and pathological images was too large, and our own dataset was already very large; therefore, we chose to train from scratch.

We extract the feature maps of the third, fourth and fifth convolution layers of the VGG16 network, and then use average pooling to compress the original 56*56*256, 28*28*512 and 14*14*512 feature maps into 1*256, 1*512 and 1*512 vectors. Then, the three vectors are concatenated into a 1280-dimension vector, which was used as the richer feature representation of the pathological image. The specific fusion process is shown in Fig. 4. Note that our method of extracting richer multilevel features can be easily adapted to any state-of-the-art CNN. In addition, we have also attempted to extract all feature maps of the VGG16 (detailed in the experimental section), which does not provide a significant classification improvement, but it greatly increases the computation costs.

### Structured EMR data feature representation

To minimize the information loss for each modality before data fusion, we need to increase the low-dimensional modality instead of reducing the high-dimensional modality before data fusion. By this way, there will be enough information in each modality before information fusion, which fulfills a prerequisite for more sufficient information fusion. Meanwhile, in the practical application scenarios of hospitals, missing data are inevitable. Therefore, it is necessary to propose new methods to deal with this problem in a targeted way.

We use denoising autoencoder as a way to increase the data dimension to achieve the goal of improving the effectiveness of the data fusion and the robustness of the methods. Autoencoder is a kind of unsupervised machine learning model whose output data are a reconstruction of the input data, and it is often used for dimensionality reduction. However, there has been little use of autoencoders or their variants to increase the dimension of data. Denoising autoencoder shares the same structure as autoencoder, but the input data are noisy versions of the raw data. To force the encoder and decoder to learn more robust feature representation and prevent them from simply learning the identity function mapping, the autoencoder is trained to reconstruct the raw input data from its noisy versions.

**Fig. 5:**
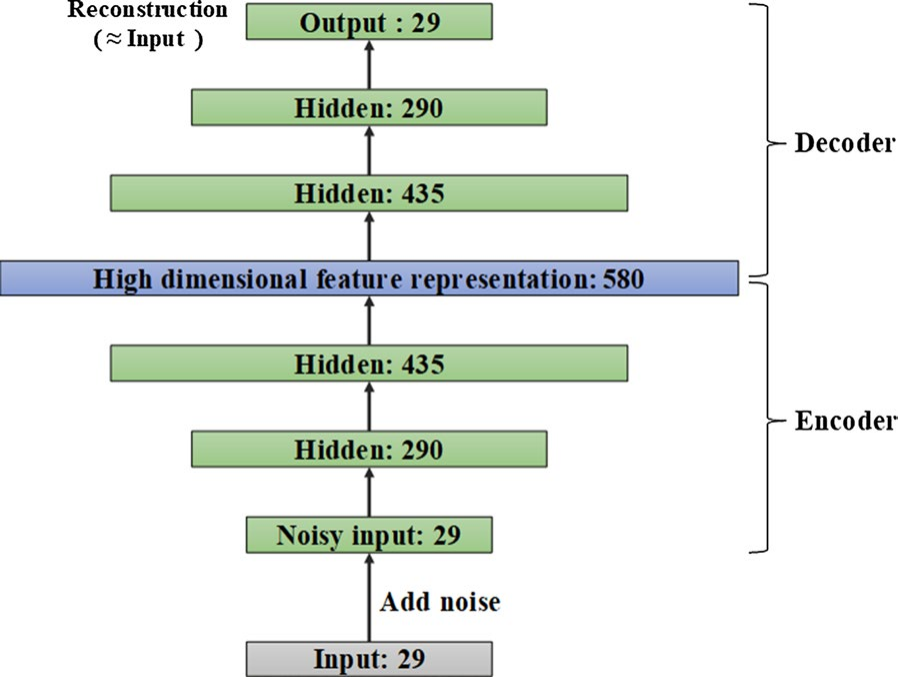
Topology structure diagram of denoising autoencoder

Because the features that we extracted from the structured data are 29 dimensions, there are 29 nodes in the input layer of the denoising autoencoder. After many experiments, the numbers of network nodes of the denoising autoencoder that we finally adopted are 29, 290, 435, 580, 435, 290 and 29. The specific network structure diagram is shown in Fig. 5.

Generally, the number of hidden layer nodes of the autoencoder is smaller than that of the input layer. In other words, during the training process, the autoencoder is forced to learn the internal features of data, and it obtains a reduced-dimensional representation of the input data. In this case, we can only use mean squared error (MSE) as the loss function. However, to increase the dimension of data, we set the number of nodes in the hidden layer to be larger than the number of nodes in the input layer. Furthermore, we also want to obtain the representative features in the input data. This approach requires us to add additional regularizations to prevent the model from only learning the input-to-output identity function mapping and not knowing any useful information about the data distribution.

Therefore, we add the L1 norm as a regular term to the loss function. The L1 regularization term makes the parameters of the model be zero as much as possible, thus increasing the sparsity of the network. Finally, the total loss function *L*(*w*) of our autoencoder is as follows:

1 $$L\left( w \right) = \frac{1}{N}\mathop \sum \limits_{i = 1}^{N} \left( {f\left( {x_{i} ;w} \right) - y_{i} } \right)^{2} + \lambda \left\| {w_{1} } \right\| ,$$

where ‘*x*_*i*_’ represents the original input data, ‘*y*_*i*_’ represents the reconstructed data of the autoencoder, ‘*λ*’ represents the regularization coefficient, and ‘*w*’ represents the parameter to be learned by the model.

In the training process, instead of adding noise to the original input data, we partially discard some features of the input samples. Specifically, we let the input data D_raw_ pass through a dropout layer with a drop rate equal to 0.2, and the output data D_noise_ after the dropout operation as the noise-added data. Then, (D_raw_, D_noise_) is used as a pair of training sample to train the denoising autoencoder until the network converges. This strategy generalizes our method to make an accurate prediction with absent of part structured data. Moreover, the experimental results show that the training method for partially missing data can not only alleviate the impact of missing data, but also reduce the risk of overfitting the entire algorithm model.

### Multimodal data fusion

For each pathological image, we extracted a vector of 1280 dimensions as the feature representation. Meanwhile, in order to ensure that different modalities can be fused at the same order of magnitude, 29 dimensions of structured EMR data are increased to 580 dimensions as the feature representation. After extracting the richer feature representation of each modality, we can combine the data of different modalities. The 580-dimensional feature representation of structured EMR data was concatenated with the 1280-dimensional feature representation vector that was extracted from a pathological image to form an 1860-dimensional vector. This vector then progresses through the next three fully connected layers to obtain a classification result of benign or malignant breast cancer. The three fully connected layers have 500, 100, and 2 nodes, respectively.

Finally, we introduce the training strategy of the entire richer fusion network. The entire network is divided into three phases for training. First, we independently train the denoising autoencoder. Then, we trained the VGG16 for the classification task of benign and malignant pathological images until it converges. Finally, we fixed the model parameters of the denoising autoencoder and the VGG16 network and trained the fully connected neural network of the data fusion part. We searched for the corresponding EMR according to the index of each pathological image. Then, pairs of pathological images and structured EMR data were sent to the network for training.

## Results and discussion

In this section, we will evaluate the performance of our proposed richer fusion network on our released dataset. We randomly selected 80% of the dataset to train the model, and the remaining 20% was used for testing. We mainly use the average accuracy to evaluate the performance of our method. Apart from the average accuracy, the performance of a classifier can be further evaluated using the ROC curve and AUC. Assuming a patient belongs to the class, classify it as positive, otherwise classify it as negative, and the accuracy can be defined as follows: Accuracy = (TP + TN)/(TP + TN + FP + FN), where: TP (TN) = Number of True Positive (True Negative) classified patients; FP (FN) = Number of False Positive (False Negative) classified patients.

### Accuracy comparison with previous methods

The performance of our proposed method is shown in Table 3. We compared the accuracy with those of the state-of-the-art methods. For the benign and malignant breast cancer classifications, our method achieved an average accuracy of 92.9%. We only compare our method to the methods that use single-mode pathological images. Since the structured data in clinical EMR only plays a supporting role, pathological images are the gold standard for final diagnoses in clinical practice. Therefore, few existing studies have only used the structured data in clinical EMR to diagnose breast cancer. Because some of the previously published papers reported 2-class classification, and others reported 4-class classification, to provide a comprehensive comparison, we compared the accuracy of our methods with those of all the methods.

**Table 3:** Comparison of accuracy with previous methods

Methods	Accuracy (%)
Bayramoglu et al. (two-class) [4]	83
Spanhol et al. (two-class) [25]	85
Araujo et al. (two-class) [26]	83.3
Rakhlin et al. [27]	87.2
Vang et al. [28]	87.5
Golatkar et al. [29]	85
Awan et al. [30]	83
Cao et al. [31]	87.1
Aresta et al. (BACH contest) [23]	87
Our proposed	92.9

### Accuracy comparison using different dimensional fusion methods

For our proposed method of integrating low-dimensional structured data and high-dimensional unstructured data using different strategies, we compared their overall performance using the average classification accuracy in Table 4. When only structured EMR data were used, the classification accuracy was not very high at only 78.5% on the test set. This is a reasonable result. Because the structured data from clinical EMR only plays a supporting role, pathological images are the gold standard for final diagnoses in clinical practice. Meanwhile, the use of structured EMR data alone is the only case that causes overfitting. Since the amount of structured EMR data is relatively small, the amount of pathological image data is large, especially after the data enhancement of the pathological image.

**Table 4:** Comparison of accuracy using different dimensional fusion of structured data and pathological image

Method	Accuracy (validation) (%)	Accuracy (test) (%)
Structured data only (29D)	90.3	78.5
Pathological image only (VGG16)	84.5	83.6
Structured data (29D) + Pathological image (FC-29D)	88.2	87.9
Structured data (29D) + Pathological image (FC-1280D)	88.6	85.2
Structured data (29D*10) + Pathological image (FC-1280D)	89.2	88.6
Structured data (29D*20) + Pathological image (FC-1280D)	93.3	91.1
Structured data (29D*30) + Pathological image (FC-1280D)	92.5	89.2
Structured data (29D*40) + Pathological image (FC-1280D)	89.5	88.7

Due to the rapid growth of deep learning in the field of computer vision, the accuracy of breast cancer classification using only pathological images has been relatively high. Therefore, when we used the VGG16 to classify pathological images, we also achieved a relatively high accuracy of 83.6%.

Although the classification accuracy when using only structured EMR data is not high, we can leverage the structured EMR data to improve the accuracy of pathological image classification. When integrating the feature representation of 29-dimensional structured EMR data and 29-dimensional pathological images, we achieved an average accuracy of 87.9%. We added a fully connected layer with 29 nodes at the end of the VGG network to obtain a 29-dimensional feature representation of the pathological images.

Further, we compared two different fusion methods for high-dimensional pathological images and low-dimensional structured EMR data. The experimental results show that it is better to first reduce the feature representation to 29-dimensional vector extracted from the last fully connected layer of the VGG16, and then fuse it with the 29-dimensional structured EMR data. This strategy is better than directly integrating 29-dimensional structured EMR data with the 1280-dimensional feature representation of pathological images. This observation is because the dimension of the 29-dimensional vectors is too low compared to the 1280-dimensional vectors. In this fusion, the high-dimensional vectors completely overwhelm the low-dimensional ones. It should be noted that the feature representation of the 1280-dimensional pathological images refers to the vector that is extracted from the end of the VGG16 with a fully connected layer with 1280 nodes and does not refer to the richer feature representation that is obtained by extracting multiple convolutional layers of the VGG16. The purpose of this is to better control the variables and clarify the comparison experiments.

Finally, after attempting to amplify the different scaled feature dimensions using the denoising autoencoder, 20-fold amplification of the structured EMR data yielded the best results. When the structured data are amplified 10 times, the overall accuracy increased, but not to the maximum. The reason is that the amplification works, but it does not reach saturation. However, if the low-dimensional structured data is amplified too much, the accuracy will decrease. One possible explanation is that the most important information still exists in the pathological images, and the excessive amplification of the influence of structured EMR data will make the fusion worse. See Table 4 for a detailed comparison of experimental results with different hyperparameter settings.

### ROC comparison using different feature representation of pathological image

**Fig. 6:**
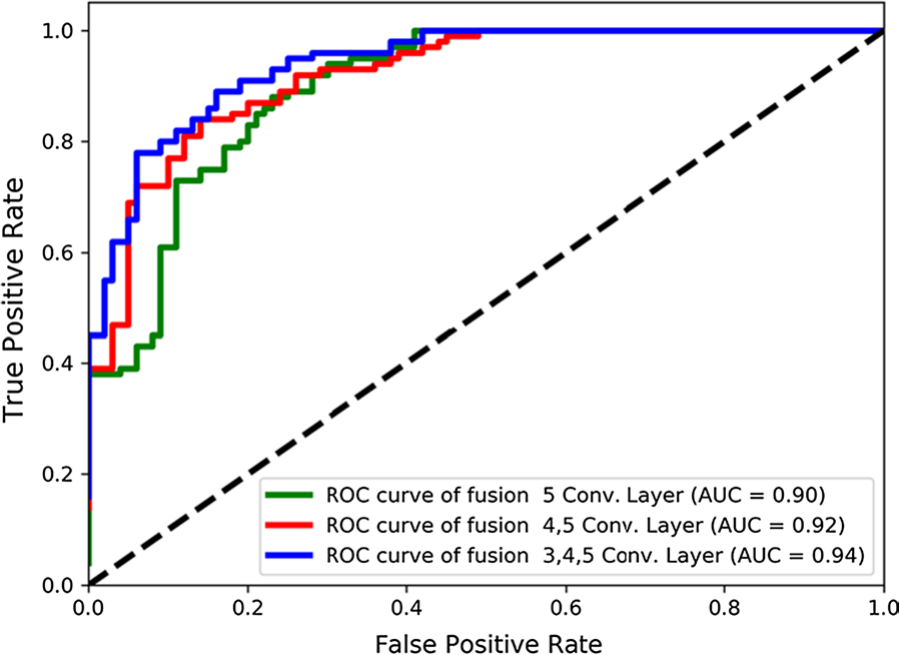
ROC curves and AUC area were compared integrating different convolutional layers of VGG16. Except for the different, the other experimental settings were controlled as follows: Structured data (denoising autoencoder, 580D, 0.2 missing rate, L1 regularization) + Fully connected layers (500, 100, 2)

We can use the richer multilevel features that were extracted from the different convolutional layers of the VGG16 to further improve the classification accuracy of our method. After trying to fuse different levels of convolutional layers, we obtained our best model by integrating structured EMR data and the third, fourth and fifth convolutional layers that were extracted from the VGG16 network (1860 dimensions). The area under the curves (AUCs) [32] using different fusion methods based on the receiver operating characteristic (ROC) analysis is shown in Fig. 6.

As seen from the experimental results in Fig. 6, when only the last convolutional layer is used (i.e., the fifth convolutional layer), the final AUC of the entire network is 0.90. Then, fusing the fourth and fifth convolutional layers achieved the AUC of 0.92. Finally, when the third, fourth, and fifth convolutional layers are integrated, the entire network achieves the best results, with an AUC of 0.94. We also tried to fuse all the five convolutional layers of VGG16. However, when the first and second convolutional layers were also fused, the experimental results were unstable and were not significantly improved, but they significantly increased the computation costs. Therefore, we did not show the comparison results in the figure.

### Accuracy comparison using different dimension raising methods

**Fig. 7:**
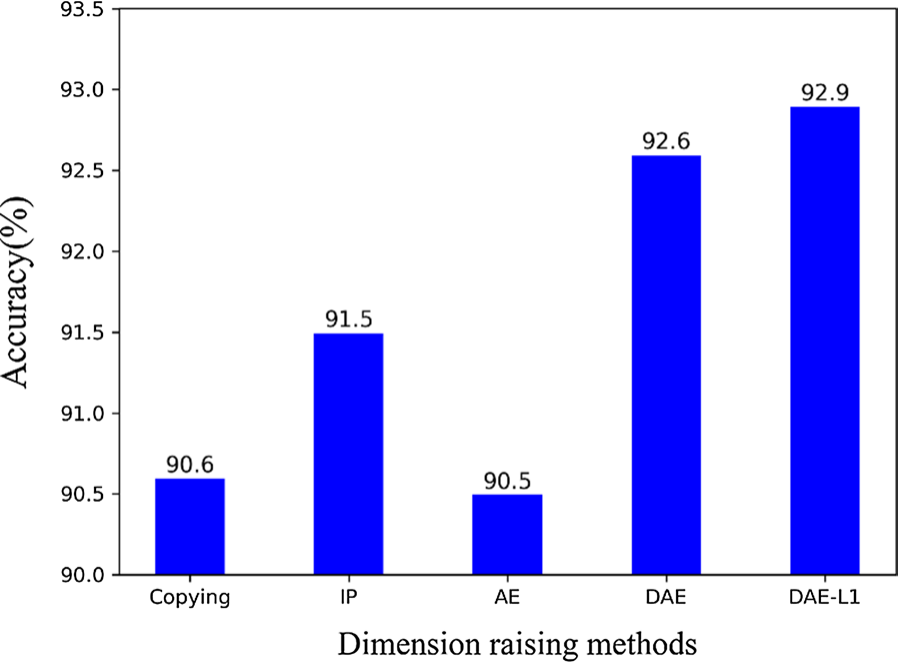
Comparative analysis using different dimension raising methods: Copying features, Interactive and Polynomial features (IP), AutoEncoder features (AE), Denoising AutoEncoder features (DAE), and Denoising AutoEncoder with L1 regularization features (DAE-L1). Except for the different dimension raising methods, the other experimental settings were controlled as follows: Structured data (580D) + Pathological image (3, 4, 5 convolution layer, 1280D) + Fully connected layers (500, 100, 2)

In this section, we compared different dimension raising methods to prove the effectiveness of using the denoising sparse autoencoder. Fig. 7 shows the overall classification accuracies that were obtained by different methods on the same dataset. In the overall model, except for the use of different dimension raising methods, the other experimental settings were controlled as follows: Structured data (580) + Pathological image (3, 4, 5 convolution layer, 1280D) + Fully connected layers (500, 100, 2).

In addition to the simple method of copying a certain percentage of the original data, the currently popular method of increasing the data dimension is to use the interactive features and polynomial features. For the original 29-dimensional feature in EMR, when the second-order interactive and polynomial features are used, the feature dimension rises to 465 dimensions. To better control the variables, we also copied a certain percentage of this 465-dimensional vector, which increased it to 580 dimensions. It can be seen from the experimental results that the interactive and polynomial features are better than copying features, and the overall accuracy of the model is improved by 0.9%. Because the interactive and polynomial features obtain a higher dimension and the mutual relationship term of the original feature by adding some nonlinear transformations of the input data, the data after the dimension raising are more representative.

The accuracy of the method using the autoencoder feature is almost the same as that of copying features. Our explanation for the appearance of such a result is that the autoencoder is generally used for the dimension reduction task. For this task, because of the need to learn the low-dimensional representation of the input data, this constraint on the network architecture forces the encoder and decoder to not only learn an input to output identity function mapping. However, our goal is to use the autoencoder to increase the dimensions of the input data. Since there are many redundant nodes in the middle layers, this makes it possible for the autoencoder to simply copy the input to the output. That is, only an identity function mapping is learned.

The denoising autoencoder achieved better results than the three methods described earlier. To force the encoder and decoder to learn more robust feature representation and prevent it from simply learning the identity function mapping, the denoising autoencoder is trained to reconstruct the raw input data from a missing version of it. Thus, the denoising autoencoder must learn to recover missing data to reconstruct the input information that has not been corrupted by the missing data. Further, we continue to increase the constraints. When we add the L1 regularization to the denoising autoencoder, our overall model achieves the best results. This result is because the L1 regularization term makes the parameters of the model to be zero as much as possible, thus increasing the sparsity constraint of the network. The denoising autoencoder combined with the L1 regularization is also called the denoising sparse autoencoder.

Finally, it should be emphasized that the denoising autoencoder is naturally suitable for solving the problem of partially missing data, which often happens in real scenarios. This is a problem that cannot be solved by copying features or by using interactive and polynomial features or autoencoder features.

### Accuracy comparison using different missing rates

**Fig. 8:**
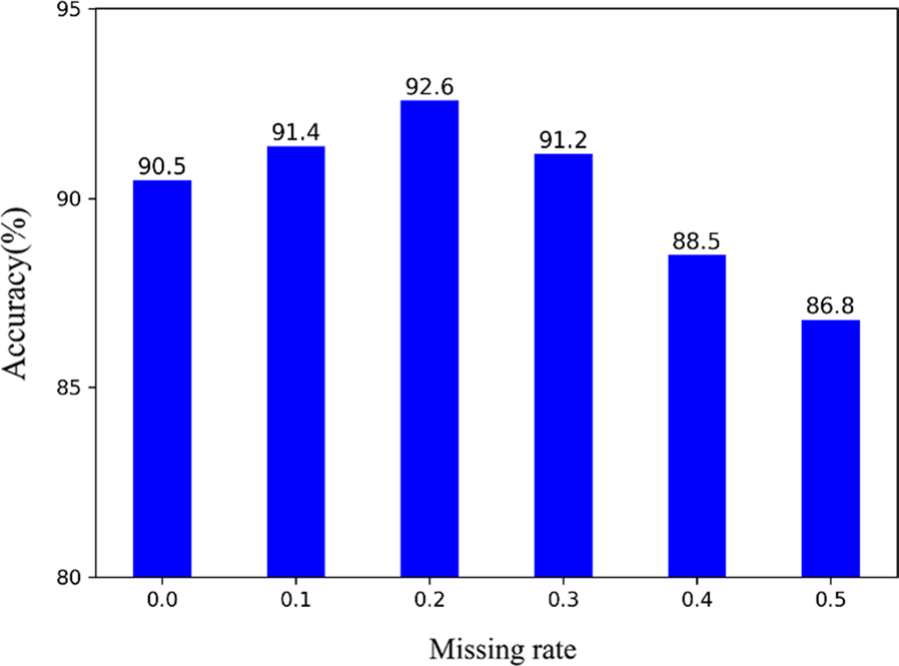
Comparative analysis using different missing rate. Except for the different missing rate, the other experimental settings were controlled as follows: Structured data (denoising autoencoder, 580D) + Pathological image (3, 4, 5 convolution layer, 1280D)

We also compared the performance of the denoising autoencoder using different missing data rates for the original input data. In the training process, instead of adding noise to the original input data, we partially discard some features of the input samples. Specifically, we let the input data pass through a dropout layer at a certain drop rate, and the output data after the dropout operation as the missing data. The missing data are completely random. When the structured data are not missing at all, the situation is the same as the experimental setting in section 5.4: Structured data (denoising autoencoder, 580D) + Pathological image (3, 4, 5 convolution layer, 1280D). It can be seen from the experimental results (as shown in Fig. 8) that the average accuracy is the highest when the missing rate is 0.2. When the missing rate is too high, too much information is lost in the structured data. At this time, it is difficult to reconstruct the original input data from the noisy input data.

## Conclusions

In this paper, we proposed a new richer fusion network for breast cancer classification based on pathological image and structured EMR data. Or from another perspective, we utilized a new method to integrate highly heterogeneous data to leverage structured EMR data to improve pathological image classification accuracy. Through comprehensive evaluation and comparison, our proposed method is superior to the state-of-the-art method. Therefore, the application of breast cancer automatic classification algorithm in clinical practice becomes possible. Due to the generality of the proposed fusion workflow, it can be straightforwardly extended to other fusion of structured data and unstructured data.

Meanwhile, we released a new dataset with pathological images and pairwise multiple types of features extracted from EMR for breast cancer classification research. We hope this dataset can be used as a benchmark to promote the wider application of machine learning methods in the field of breast cancer research.

## Data Availability

We have released the dataset that support the findings of this study, which can be downloaded publicly from http://ear.ict.ac.cn/?page_id=1663.

## Abbreviations

EMR: Electronic medical record
CNN: Convolutional neural network
VGG: Visual Geometry Group
WSI: Whole slide image

## References

[1] Siegel RL, Miller KD, Dvm AJ (2019). Cancer statistics, 2019. CA Cancer J Clin.

[2] Litjens G, Kooi T, Bejnordi BE, Setio AAA, Ciompi F, Ghafoorian M, van der Laak J, van Ginneken B, Sanchez CI (2017). A survey on deep learning in medical image analysis. Med Image Anal.

[3] Spanhol FA, Oliveira LS, Petitjean C, Heutte L (2016). A dataset for breast cancer histopathological image classification. IEEE Trans Biomed Eng.

[4] Bayramoglu N, Kannala J, Heikkilä J. Deep learning for magnification independent breast cancer histopathology image classification. In: International conference on pattern recognition. 2017. p. 2440–5.

[5] Shen D, Wu G, Suk HI (2017). Deep learning in medical image analysis. Annu Rev Biomed Eng.

[6] Zhang Q, Yang LT, Chen Z, Li P (2018). A survey on deep learning for big data. Information Fusion.

[7] Yao J, Zhu X, Zhu F, Huang J. Deep correlational learning for survival prediction from multi-modality data. In: International conference on medical image computing and computer-assisted intervention. Springer; 2017. p. 406–14.

[8] Mobadersany P, Yousefi S, Amgad M, Gutman DA, Barnholtz-Sloan JS, Velázquez Vega JE, Brat DJ, Lad C (2018). Predicting cancer outcomes from histology and genomics using convolutional networks. Proc Natl Acad Sci USA.

[9] Xu T, Zhang H, Huang X, Zhang S, Metaxas DN. Multimodal deep learning for cervical dysplasia diagnosis. In: International conference on medical image computing and computer-assisted intervention. Springer; 2016. p. 115–23.

[10] Khaleghi B, Khamis A, Karray FO, Razavi SN (2013). Multisensor data fusion: a review of the state-of-the-art. Inf Fusion.

[11] Ramachandram D, Taylor GW (2017). Deep multimodal learning: a survey on recent advances and trends. IEEE Signal Process Mag.

[12] Zhang Z, Chen P, Sapkota M, Yang L. Tandemnet: Distilling knowledge from medical images using diagnostic reports as optional semantic references. In: International conference on medical image computing and computer-assisted intervention. Springer; 2017. p. 320–8.

[13] Wang X, Peng Y, Lu L, Lu Z, Summers RM. Tienet: Text-image embedding network for common thorax disease classification and reporting in chest x-rays. In: Proceedings of the IEEE conference on computer vision and pattern recognition. 2018; p. 9049–58.

[14] Sermanet P, Chintala S, LeCun Y. Convolutional neural networks applied to house numbers digit classification. arXiv preprint arXiv:1204.3968. 2012.

[15] Liu Y, Cheng M-M, Hu X, Wang K, Bai X. Richer convolutional features for edge detection. In: Proceedings of the IEEE conference on computer vision and pattern recognition. 2017. p. 3000–9.

[16] Bertasius G, Shi J, Torresani L. Deepedge: A multi-scale bifurcated deep network for top-down contour detection. In: Proceedings of the IEEE conference on computer vision and pattern recognition. 2015. p. 4380–4389.

[17] Xie S, Tu Z (2017). Holistically-nested edge detection. Int J Comput Vision.

[18] Xing F, Xie Y, Su H, Liu F, Yang L (2018). Deep learning in microscopy image analysis: a survey. IEEE Trans Neural Netw Learn Syst.

[19] Bengio Y (2009). Learning deep architectures for AI. Found Trends Mach Learn.

[20] Vincent P, Larochelle H, Bengio Y, Manzagol P-A. Extracting and composing robust features with denoising autoencoders. In: Proceedings of the 25th international conference on machine learning, Helsinki, Finland. ACM; 2008. p. 1096–1103.

[21] Ngiam J, Khosla A, Kim M, Nam J, Lee H, Ng AY. Multimodal deep learning. In: Proceedings of the 28th international conference on international conference on machine learning, Bellevue, Washington, USA. Omnipress; 2011. p. 689–96.

[22] Russakovsky O, Deng J, Su H, Krause J, Satheesh S, Ma S, Huang Z, Karpathy A, Khosla A, Bernstein M (2015). ImageNet large scale visual recognition challenge. Int J Comput Vision.

[23] Aresta G, Araújo T, Kwok S, Chennamsetty SS, Safwan M, Alex V, Marami B, Prastawa M, Chan M, Donovan M (2019). BACH: grand challenge on breast cancer histology images. Med Image Anal.

[24] Simonyan K, Zisserman A. Very deep convolutional networks for large-scale image recognition. arXiv preprint arXiv:1409.1556. 2014.

[25] Spanhol FA, Oliveira LS, Petitjean C, Heutte L. Breast cancer histopathological image classification using convolutional neural networks. In: International joint conference on neural networks. 2016. p. 717–726.

[26] Araújo T, Aresta G, Castro E, Rouco J, Aguiar P, Eloy C, Polónia A, Campilho A (2017). Classification of breast cancer histology images using convolutional neural networks. PLoS ONE.

[27] Rakhlin A, Shvets A, Iglovikov V, Kalinin AA. Deep convolutional neural networks for breast cancer histology image analysis. In: International conference image analysis and recognition. 2018. p. 737–44.

[28] Vang YS, Chen Z, Xie X. Deep learning framework for multi-class breast cancer histology image classification. In: International conference image analysis and recognition. 2018. p. 914–22.

[29] Golatkar A, Anand D, Sethi A. Classification of breast cancer histology using deep learning. In: International conference image analysis and recognition. Springer; 2018. p. 837–844.

[30] Awan R, Koohbanani NA, Shaban M, Lisowska A, Rajpoot N. Context-aware learning using transferable features for classification of breast cancer histology images. In: International conference image analysis and recognition. 2018. p. 788–95.

[31] Cao H, Bernard S, Heutte L, Sabourin R. Improve the performance of transfer learning without fine-tuning using dissimilarity-based multi-view learning for breast cancer histology images. In: International conference image analysis and recognition. Springer; 2018. p. 779–787.

[32] Jiao Y, Du P (2016). Performance measures in evaluating machine learning based bioinformatics predictors for classifications. Quant Biol.

